# Unraveling effects of anti-aging drugs on *C. elegans* using liposomes

**DOI:** 10.1007/s11357-023-00800-x

**Published:** 2023-05-04

**Authors:** Aihan Zhang, Kuei Ching Hsiung, Carina C. Kern, Yuting Wang, Anna L. Girtle, Nuo Xu, David Gems

**Affiliations:** Institute of Healthy Ageing, and Research Department of Genetics, Evolution and Environment, University College London, London, WC1E 6BT UK

**Keywords:** Aging, *C. elegans*, Drug, Infection, Liposomes

## Abstract

Liposome-mediated delivery is a possible means to overcome several shortcomings with *C. elegans* as a model for identifying and testing drugs that retard aging. These include confounding interactions between drugs and the nematodes’ bacterial food source and failure of drugs to be taken up into nematode tissues. To explore this, we have tested liposome-mediated delivery of a range of fluorescent dyes and drugs in *C. elegans*. Liposome encapsulation led to enhanced effects on lifespan, requiring smaller quantities of compounds, and enhanced uptake of several dyes into the gut lumen. However, one dye (Texas red) did not cross into nematode tissues, showing that liposomes cannot ensure the uptake of all compounds. Of six compounds previously reported to extend lifespan (vitamin C, *N*-acetylcysteine, glutathione (GSH), trimethadione, thioflavin T (ThT), and rapamycin), this effect was reproduced for the latter four in a condition-dependent manner. For GSH and ThT, antibiotics abrogated life extension, implying a bacterially mediated effect. With GSH, this was attributable to reduced early death from pharyngeal infection and associated with alterations of mitochondrial morphology in a manner suggesting a possible innate immune training effect. By contrast, ThT itself exhibited antibiotic effects. For rapamycin, significant increases in lifespan were only seen when bacterial proliferation was prevented. These results document the utility and limitations of liposome-mediated drug delivery for *C. elegans*. They also illustrate how nematode-bacteria interactions can determine the effects of compounds on *C. elegans* lifespan in a variety of ways.

**Supplementary Information:**

The online version contains supplementary material available at 10.1007/s11357-023-00800-x.

## Introduction

The short-lived nematode *Caenorhabditis elegans* is in many respects a highly convenient model organism for studying the biology of aging. One might therefore have thought that it would be ideally suited to identify and study drugs that slow the aging process, yet in certain respects it is not. This is for several reasons, enumerated in detail in a recent review [1], partly relating to problems with drug uptake into the worm.

In the laboratory *C. elegans* are usually maintained on the surface of agar plates with a live bacterial food source (*Escherichia coli*). Under these conditions, lifespan is limited by bacterial infection that occurs in senescent worms; consequently, treatments that prevent such infection (such as use of antibiotics, or UV irradiation of bacteria) extend lifespan [2-4]. Thus, drugs that reduce the pathogenicity of *E. coli* can increase *C. elegans* lifespan; such anti-bacterial effects can confound tests for direct inhibition of nematode aging.

While confounding effects of infection can be prevented with antibiotics or UV irradiation, other effects of bacteria on lifespan remain. *E. coli* subjected to treatments that prevent their proliferation do not necessarily cause cell death, and treatments that cause actual death often render *E. coli* unable to sustain *C. elegans* growth [5]. This may reflect the close relationship between *C. elegans* and its intestinal microbes, where the latter function as a microbiome that is required for normal nematode growth [5, 6]. For example, life-extending effects of the anti-diabetic drug metformin are dependent upon the presence of live bacteria but not bacterial proliferation [7]. A further complication is that *E. coli* may biotransform the drug before it reaches the worm, though such effects can provide a useful model for understanding how the microbiome influences drug action [8].

Drug-bacteria interactions can be excluded by culturing *C. elegans* on an axenic nutrient medium (i.e., with no bacteria present). Unfortunately, *C. elegans* not only grow poorly on axenic medium, but also exhibit extended lifespan [9]. This appears to be due to some form of dietary restriction (DR) effect, in addition to the absence of bacterial infection [10].

Thus, bacterially mediated effects of drugs affecting *C. elegans* lifespan can be difficult to exclude. Heat-killing of *E. coli* is sometimes proposed as a solution, but for the reasons listed above, it cannot be: if it fully kills *E. coli*, *C. elegans* will experience DR; if it does not, bacterially mediated effects will not be excluded. Autoclaved *E. coli* does not support normal reproduction and aging in *C. elegans* [5]. However, a recently described possible solution is the use of *E. coli* killed using paraformaldehyde, on which *C. elegans* grow well and, surprisingly, exhibit a normal (non-extended) lifespan, despite abrogation of infection [11].

A second major issue is that many drugs administered to *C. elegans* fail to cross efficiently from the lumen of the intestine into the tissues. In a key study of 1000 drug-like small molecules, less than 10% accumulated to concentrations greater than 50% of that present in the medium [12]. Possibly, the reticence of *C. elegans* to take up compounds reflects the hostile chemical environment in which it dwells in the wild [13], where defense against chemical attack from competing organisms is important for survival.

One potential means to mitigate these various problems with *C. elegans* as a model to investigate anti-aging drugs is liposome-mediated drug delivery. The use of liposomes is a form of nanotechnology with increasingly wide medical applications [14], as in the recent use of the liposome-delivered Pfizer-BioNTech and Moderna mRNA vaccines to protect against COVID-19. A liposome is a tiny, spherical liquid-filled bubble, bounded by a lipid bilayer. They can function as vehicles for drug delivery, and liposome fusion with plasma membranes facilitates drug entry into cells. Preparations of compound-loaded liposomes remain stable at 4 °C for up to 4 days.

Encapsulation within liposomes should reduce the effects of drugs on *E. coli*, and the biotransformation of drugs by *E. coli*. Liposomes might also aid the transport of drugs across the apical membrane of *C. elegans* intestinal cells, from the gut lumen into the cell interior. Moreover, the particulate nature of liposomes should aid the transport of drugs into the intestinal lumen. This is because the terminal bulb of the *C. elegans* pharynx functions by concentrating particulate matter (particularly bacterial cells) and then transporting them back into the intestinal lumen [15-17].

Liposome-mediated delivery can reduce the amount of drug needed for trials, thereby reducing drug costs. A standard approach is to add the drug to the agar plate to a given concentration; for example, standard 60-mm and 30-mm-diameter Petri dishes used for *C. elegans* culture typically contain 10 ml and 5 ml of agar, respectively. Most of the drug is inaccessible to the worm, buried in the agar. By contrast, volumes of liposomes of 25 μl [16] or 50 μl (this study) are sufficient which when laid onto the surface of bacterial lawns renders all of the drug accessible to nematodes.

It has also been reported that liposome-mediated encapsulation of axenic medium is sufficient to support normal growth of *C. elegans* [15]. This again may reflect the inability of *C. elegans* to take up nutrients supplied in purely liquid form; consistent with this, the inclusion of particulate matter in the axenic medium improves worm growth upon it [18].

It was previously shown in a groundbreaking study that liposome-mediated delivery can increase the uptake of the green fluorescent dye uranine (fluorescein) into *C. elegans* tissues, and also render several compounds (vitamin C, *N*-acetylcysteine [NAC], glutathione [GSH], and trimethadione) able to increase lifespan [16]; a recent study underscored the fact that NAC and GSH (non-liposome encapsulated) do not increase lifespan, but rather shorten it [19]. In this study, we explore further the potential for liposome-mediated drug delivery to overcome the various drawbacks of *C. elegans* as a model for studying anti-aging drugs. Our findings confirm that liposome-mediated delivery is an effective way to deliver drugs into the intestinal lumen, but not necessarily to cross the intestinal apical membrane. We also explore the extent to which several drugs previously reported to increase lifespan are dependent upon the capacity of *E. coli* to proliferate. Our findings delineate several ways in which bacterial proliferation determines how drugs affect *C. elegans* lifespan. This study should help inform experimental design in future work on anti-aging drugs in *C. elegans*.

## Methods

### Culture methods and strains

*C. elegans* maintenance was performed using standard protocols [20]. Unless otherwise stated, all strains were grown at 20 °C on nematode growth media (NGM) plates seeded with *E. coli* OP50 as a food source.

In some trials, *E. coli* OP50 bacterial lawns were treated with antibiotics to prevent infection of *C. elegans* with *E. coli*. Lawns were incubated for 48 h after inoculation to allow bacterial growth before adding the antibiotic. Eighty microliters of 500 mM carbenicillin in Milli-Q water was added topically to 60-mm-diameter plates containing ~10 ml NGM, giving a final concentration of ~4 mM carbenicillin. Worms were transferred to the plates ~12 h later.

In trials with the RNAi control strain (*E. coli* HT115 with plasmid L4440), bacteria were added to LB broth medium containing 1 mM IPTG, cultured at 37 °C overnight, and then seeded onto NGM plates.

An N2 hermaphrodite stock recently obtained from the Caenorhabditis Genetics Center was used as wild type (N2H) [21]. Strains used included DA597 *phm-2(ad597)*; LSC897 *ceh-60(lst466)*, which was kindly provided by Liesbeth Temmerman (Leuven University, Belgium); SJ4103 *zcIs14*(P*myo-3*::GFP^mt^) (GFP in body wall muscle mitochondria); and SJ4143 *zcIs17*(P*ges-1*::GFP^mt^) (GFP in intestinal cell mitochondria). Other bacterial strains included OP50-RFP, *E. coli* OP50 containing pRZT3, expressing a red fluorescent protein. pRZT3 confers tetracycline resistance and contains the DsRed gene expressed from the constitutive lac promoter [3].

### Preparation of liposomes

To create liposomes, L-α-phosphatidylcholine (L-α-PC) (Avanti Polar Lipids) was used, following an earlier protocol [16] unless otherwise stated. A few trials used 1,2-dimyristoyl-sn-glycero-3-phosphocholine (DMPC), a more stable lipid [15]. Liposomes were prepared in a laminar flow hood to avoid microbial contamination, using an Avanti Mini Extruder as previously described [16]. Lipid was kept above the phase transition temperature (70 °C) during hydration and extrusion. Unless otherwise stated, an extruder polycarbonate membrane with 100-nm-diameter pores was used to produce liposomes. Liposome extruder purification (LEP) [22] was performed using a smaller pore-size polycarbonate membrane (50-nm diameter). LEP allows the removal of non-liposome encapsulated compounds (drugs or dyes) after liposome preparation.

Liposome formation was confirmed by measurement of optical absorbance. Lipid particles in suspension cause light scattering leading to a milky opacity, while the formation of liposomes reduces light scattering and increases transparency. An Infinite 200 PRO microtiter plate reader (Tecan) was used to measure the absorption spectra before and after the extrusion process. Twenty microliters of sample was added to each well of a 96-well plate. The absorbance was calibrated by making a calibration blank with M9 buffer. Absorbance was measured at 200–950 nm.

For nematode survival trials, liposome preparations were checked for contamination by streaking the suspension onto LB plates, leaving them overnight at 37 °C, and checking for absence of growth of bacterial colonies.

### Liposome-mediated compound delivery

For liposome-mediated delivery to *C. elegans*, a small lawn of *E. coli* was used, created by adding a smaller volume (50 μl) of OP50 suspension to each plate. This was done to enable the liposome suspension to cover a larger proportion of the lawn surface, thereby reducing heterogeneity in exposure of nematodes to liposomes.

For studies of fluorescent dye uptake, worms were placed on plates seeded with 100 μl OP50, and 50 μl free or liposome-encapsulated dye was added to the lawn surface. The concentrations of the dyes used were acridine orange (green), 20 μM; Texas red, 1.6 mM; and uranine (green), 52.8 mM. Dye-treated plates were kept in a light-proof box to avoid photobleaching. After incubation with the dye, worms were subjected to a cleaning step to remove dye from their surface before imaging, using either a “wash” or “chase” protocol. The wash protocol involved placing the worms in 5 ml M9 with 1% Tween-20 (a non-ionic detergent), followed by centrifuging down (2,000 RPM for 3 min, 4 °C) and removing the supernatant; this was done three times. For the chase protocol, worms were transferred to NGM plates with OP50 and left for 30 min to crawl about (thereby leaving behind external dye) and eat (thereby flushing dye from the gut lumen). Finally, worms were imaged, anatomical distribution of dye observed, and fluorescence levels measured.

For studies of drug effects on lifespan, drug solution was added to the surface of the *E. coli* lawn, either free or liposome encapsulated, at the L4 stage, and every 2 days thereafter until day 8 of adulthood, and every 4 days after that. Where compound solubility allowed it, trials were performed such that the quantity of drug on/in a plate was the same whether or not it was liposome-encapsulated. In such cases, the concentration of the compound in the liposomes was far higher (typically 200-fold) than in the agar, facilitating drug delivery. In the case of rapamycin, lower amounts of the compound were used in liposomes due to limited compound solubility.

Hydrophilic drugs were dissolved in Milli-Q water, and hydrophobic drugs in a mix of Milli-Q water and non-polar solvent stock solution (see Table 1). To encapsulate the drug in liposomes, a mixture of the drug in solvent and 48 mg/ml lipid (final concentration) was prepared and then passed back and forth through the extruder filter. Liposomes were stored at 4 °C for up to 3 days prior to use. Before use, plates were left overnight to allow free drug to diffuse throughout the plates; this was to reduce variation in drug exposure due to differences in the degree of diffusion from the agar surface. To avoid the effects of drugs on early development, worms were placed on drug-treated plates at the pre-adult L4 stage.

**Table 1:** Drugs concentrations used in experiments unless otherwise stated in figure legends

Drug	Stock solution or liposome concentration, solvent	Volume added to 10-ml NGM plate	Final concentration
Carbenicillin	500 mM, MilliQ water	80 μl	4 mM
FUDR (5-fluorodeoxyuridine)	7.5 mM, MilliQ water	100 μl	75 µM
Glutathione (GSH)	23.4 mM, MilliQ water	50 μl	117 µM
*N*-Acetylcysteine (NAC)	2.5 mM, MilliQ water	50 μl	12.5 µM
Rapamycin			
Dose 1	2 mM, DMSO	2.5 μl	0.5 µM
Dose 2	54.7 mM, DMSO	18.3 μl	100 µM
Dose 3	54.7 mM, DMSO	91.5 μl	500 µM
Dose 4	54.7 mM, DMSO	183 μl	1 mM
Thioflavin T (ThT)			
Dose 1	5 mM, MilliQ water	50 μl	25 µM
Dose 2	10 mM, MilliQ water	50 μl	50 µM
Trimethadione	167.7 mM, MilliQ water	50 μl	838.5 µM
Vitamin C	27.3 mM, MilliQ water	50 μl	136.5 µM

The compounds used were sourced as follows: acridine orange (Invitrogen), GSH (Thermo Scientific), NAC (Sigma-Aldrich), rapamycin (Bio-Techne), Texas red (Invitrogen), ThT (Sigma-Aldrich), trimethadione (Sigma-Aldrich), uranine (Sigma-Aldrich), and vitamin C (Sigma-Aldrich).

### Microscopy

Nomarski and epifluorescence microscopy. For imaging, live worms were viewed on 2% agar pads, anesthetized with 10 μl 2 mM levamisole. For brightfield imaging using Nomarski optics, and epifluorescence imaging, we used either a Zeiss Axioskop 2 plus microscope with Hamamatsu ORCA-ER digital camera C4742-95 and Volocity 6.3 software (Macintosh version) for image acquisition or a Zeiss ApoTome.2 microscope with a Hamamatsu C13440 ORCA-Flash4.0 V3 digital camera and Zen software for image acquisition.

For trials using uranine (fluorescein) and acridine orange, green fluorescence was observed using a GFP filter, (*λ*_ex_/*λ*_em_ 450–490 nm/500–550 nm) (Filter Set 90 HE). For Texas red and RFP bacteria, red fluorescence was observed using a DsRed filter, (*λ*_ex_/*λ*_em_ 530–560 nm/590–650 nm) (Filter Set 91 HE).

A constant exposure time was maintained between samples in fluorescence intensity comparisons. Worm fluorescence was estimated as the average pixel density of the worm image area minus that of the image background. Fluorescence was quantified by manually drawing around the periphery of the worm and measuring the average pixel fluorescence intensity using ImageJ.

For imaging of agar to observe the diffusion of uranine in NGM plates, 10 μl of 2 mg/ml uranine, with or without liposomes, was added onto the surface of freshly prepared agar plates at the center of the plate. For this, fresh NGM plates (poured 2 days earlier) were used. Next, thin slices of agar (1 mm) were cut at successive time intervals post dye addition from the center of the agar using a scalpel and then transferred to microscope slides. Coverslips were then placed upon the agar slices, and the degree of diffusion away from the point of addition to the plate surface measured.

#### Confocal imaging

An inverted LSM880 microscope was used with a Plan-Apochromat 63 × 1.4 [numerical aperture (NA)] oil objective with a working distance of 0.19 mm. A 488-nm Argon laser was used for GFP excitation. Emission was recorded with an inbuilt GaAsP detector.

#### Transmission electron microscopy

To confirm the presence of liposomes, 160 μl liposome solution was sonicated for 5 min to produce a dispersed suspension. Three drops of this were then dropped onto a carbon-coated copper mesh grid (200 mesh, 3.05-mm diameter) and allowed to dry before imaging. TEM images were acquired on a JEM 2100Plus Electron Microscope (JEOL, Japan) operated at 200-kV acceleration voltage.

### Use of fluorescent *E. coli* to visualize pharyngeal infection

OP50-RFP was cultured overnight at 37 °C in LB containing tetracycline (25 μg/ml). Bacteria were then washed 3 times using an M9 buffer to remove the tetracycline and inoculated onto NGM plates 2 days prior to use. L4-stage worms were transferred from plates seeded with OP50 onto plates containing OP50-RFP and then transferred daily until the time of imaging. Fluorescence within pharyngeal tissue (i.e., bacterial infection) was imaged at 630 × magnification using a Zeiss Axio Imager Z2 microscope, with Filter Set 90 and 91 HE LED. Images were scored by eye into 4 categories: no red fluorescence (uninfected); red puncta present (sites of bacterial invasion within pharyngeal tissue that correspond with low levels of infection); red fluorescence throughout the pharyngeal tissue (heavily infected); and dead with widespread red fluorescence (P death). For scoring, bacteria in the lumen of the pharynx were ignored.

### Analysis of mitochondrial morphology

SJ4143 *zcIs17* (P*ges-1*::GFP^mt^) and SJ4103 *zcIs14* (P*myo-3*::GFP^mt^) were exposed to drugs for 24 h starting at the L4 stage. Live, 1-day-old adults were then mounted onto slides and immediately imaged using confocal microscopy. Mitochondrial dynamics were assessed by measuring elongation, area, and connectivity, as previously described [23], using Fiji software (NIH) [24].

### Measurement of drug effects on bacterial growth

Measurement of bacterial growth using optical density (OD). A single *E. coli* OP50 colony was picked and cultured in OP50 media overnight in a shaking incubator (37 °C). The culture was then diluted in OP50 media to a concentration of 1/1000 and the OD 500 was measured. Next drugs were added to concentrations as described (Table 1). For ThT, the concentration of free, non-liposome encapsulated drug was quantified using its yellow color. For this, LEP was performed to collect the liquid surrounding the liposomes, and ThT concentration measured using spectrometry, and compared to a calibration curve (see “Results” section for calibration curve; ThT *λ*_ex_/*λ*_em_ = 349 nm/454 nm). Next, the OD 500 of OP50 cultures, with or without added drugs, were measured at 0, 3, 6, and 18 h at 37 °C, using an Infinite 200 PRO microtiter plate reader (Tecan).

Measurement of bacterial growth using colony counts. Drugs were added to solid NGM media plates at the same final concentrations as specified for liquid media. *E. coli* OP50 cultured overnight in OP50 medium at 37 °C in a shaking incubator was diluted to a concentration of 1/1,000,000. One hundred microliters of this solution was then spread onto NGM plates with or without the drug, and colonies counted after 24 h (37 °C).

### Survival analysis and mortality deconvolution analysis

Nematodes were maintained at a density of 25–30 per plate and transferred daily during the egg-laying period, followed by every 6–7 days thereafter. The L4 stage was defined as day 0. Mortality was scored every 1–2 days, with worms logged as alive if they showed any movement, either spontaneously or in response to gentle touch with a worm pick.

Mortality deconvolution analysis is based on the presence of two forms of death in aging *C. elegans* cultured on proliferating *E. coli*: earlier death with an infected, swollen pharynx (P death) and later death with an atrophied pharynx (p death) [3]. Mortality deconvolution involves analysis of P and p lifespans separately. Alterations in lifespan can result from altered percentages of P (and p) deaths, and/or altered P and/or p lifespan. Deconvolved mortality statistics include each of these values. Corpses were scored by necropsy as P or p using the highest magnification of a dissecting microscope.

### Axenic culture

Nematodes were cultured using axenic medium (AXM), either in liquid culture on solid agar plates, either free or in liposomes [5, 15]. The basal suspension medium is the liquid axenic medium (AXM). AXM consists of 3% (w/v) yeast extract (for growth factors and vitamins) (Becton–Dickinson, Franklin Lake, NJ) and 3% (w/v) soy peptone (for a supply of amino acids) (Sigma-Aldrich, St. Louis, MO). These ingredients are autoclaved for 1 h for sterilization, after which a hemoglobin stock solution (Serva, Heidelberg, Germany) is added to 1% (w/v). Incubations during all AXM-related trials were conducted in darkness to avoid photodegradation of hemoglobin. Additional reagents added include 5 μg/ml cholesterol, 1 mM CaCl_2_, 1 mM MgSO_4_, and 25 mM KH_2_PO_4_ pH 6.0.

All liquid culture experiments were performed in low-binding 2-ml Eppendorf tubes with 400 μl of liquid added, along with 20–50 aseptic eggs. These were obtained from gravid *C. elegans* by treating them with a 1:2 mixture of 5N NaOH and 5% sodium hypochlorite solution for 10 min and then washing them 3×. Solutions were changed every 24 h to prevent nutrient depletion by hatched larvae; this was done near a flame to prevent contamination. For nematode culture, liposome suspension was mixed with an M9 buffer at a ratio of 1:5 unless otherwise stated.

Liposomes containing liquid AXM were prepared by vortexing a lipid solution, using methods similar to those previously described [15]. Briefly, chloroform was used to dissolve and sterilize DMPC or L-α-PC lipids to a concentration of 10 mM in a glass tube. The lipid solution was then evaporated overnight in a fume hood. This differs slightly from the published method [15], which used liquid nitrogen to evaporate off the chloroform. Next, 2 ml of AXM was added and the final solution incubated at 70–80°Cs for 30 min. Finally, the solution was gently vortexed for 1 min to produce liposomes, using a Scientific Industries Vortex-Genie 2 shaker, at setting 600 rpm.

### Statistical analysis

No statistical methods were used to predetermine the sample size. The experiments were not randomized. The investigators were not blinded to allocation during experiments and outcome assessment unless otherwise stated. All statistical tests were performed on raw data using GraphPad Prism 9.0 and JMP Pro 15 unless otherwise stated, with the specific tests and post hoc corrections performed described in the figure legends.

## Results

### Characterization and optimization of liposome-mediated compound delivery

Liposomes were prepared much as previously described [16]. Since the expected size of the liposomes produced (100–300-nm diameter) is too small to be observed using light microscopy, their presence was confirmed using several approaches: first, the change in the lipid suspension from a milky to a more transparent appearance, also detectable as a reduction in optical absorbance using a plate reader (Fig. S1A); second, by means of liposome extruder purification (LEP), to obtain a high concentration of liposomes by filtering out the buffer [22]. For this, liposomes containing the green fluorescent dye uranine (52.8 mM) were first prepared by passage through 100-nm pores. The filter was then replaced by another one with 50-nm pores, through which liposomes cannot pass, allowing free dye to be removed. After successive addition of dye-free wash, uranine, identified by its absorbance peak of 490 nm using a plate reader, remained in the syringe barrel (Fig. S1B). This is consistent with the presence of liposomes, which have trapped the uranine thereby preventing its passage through the filter. Third, TEM imaging of putative liposome suspensions confirmed the presence of liposomes (Fig. S1C).

To feed liposomes to *C. elegans*, a small volume (50 μM) of liposome suspension was pipetted onto the surface of a small *E. coli* lawn. By this means, the nematodes ingest the liposomes along with their bacterial food source.

**Fig. 1:**
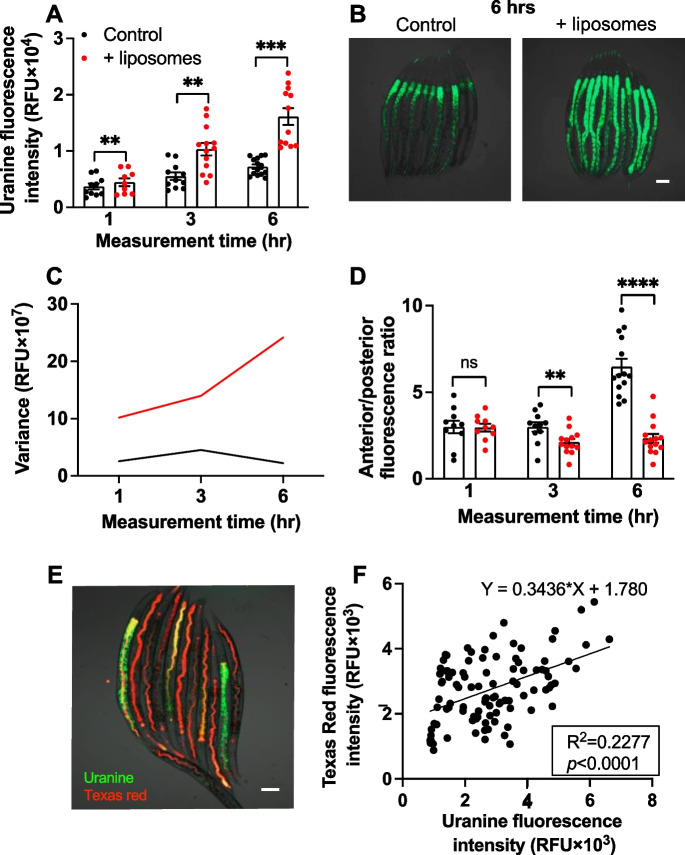
**A** Liposome encapsulation improves uranine uptake by *C. elegans*. **B** Liposome encapsulation improves compound uptake in the posterior intestine. Scale bar, 100 μm. **C** Liposome encapsulation increases variance in dye uptake (from the same dataset as **A**). **D** Liposome encapsulation significantly increased the proportion of dye in the posterior of the intestine at 6 h. **A**, **D** Mean ± S.E.M, by one-way ANOVA (Šidák correction). **E** Green and red fluorescence after serial exposure to uranine and Texas red in liposomes. Scale bar, 100 μm. **F** Green and red fluorescence levels differ significantly (*p* < 0.0001), and R^2^ is only 0.2277, i.e., only ~20% of the variation in the data is explained by differences between individual worms. Combined data of 3 trials displayed; for individual trials, see Fig. S3. The results imply that increased heterogeneity in dye uptake due to liposome-mediated delivery reflects increased heterogeneity in exposure to dye, rather than intrinsic differences between individual nematodes. **p < 0.01, ***p < 0.001

Liposome encapsulation of uranine was found to markedly increase dye uptake by *C. elegans*, as previously observed [16, 25]. Increases of 86.9% and 108.7% were seen after 3 h and 6 h of exposure, respectively (Fig. 1A). After exposure to free, non-liposome-encapsulated dye, green fluorescence was largely concentrated in the anterior half of the intestine. Liposome encapsulation significantly increased the proportion of dye in the posterior half of the intestine at 6 h (Fig. 1B, D). Thus, liposome encapsulation improves the delivery of compounds to the entire length of the intestine.

Liposome encapsulation caused an increase in the variance of dye uptake levels (Fig. 1A, C). This might be due to heterogeneity in exposure to liposomes, due, e.g., to the heterogeneous distribution of liposomes on the bacterial lawn surface, or differences in the time spent by individual nematodes on and off the bacterial lawn. Alternatively, it could be that intrinsic differences between individual worms cause some to take up more dye than others (due, e.g., to differences in feeding behavior or permeability to dye uptake).

To distinguish between these two possibilities, worms were placed first on plates with uranine and then transferred to plates with a different dye, Texas red (liposome-encapsulated in both cases). Relative levels of green and red fluorescence in individual worms were then measured. If increased dye uptake variance was due wholly to heterogeneity in exposure to liposomes, no correlation between levels of the two dyes would be expected. By contrast, intrinsic differences in dye uptake between worms would lead to a positive correlation between levels of the two dyes. In fact, a slight but significant correlation was seen in 3 trials with an R^2^ (summed data) of only ~0.2 (*p* < 0.0001; Figs. 1E, F and S2). This suggests that inter-individual variation in dye uptake is predominantly due to differences in quantity of liposomes that worms encounter, while around one-fourth of the variation can be accounted for by intrinsic variation in how well animals take up dye.

We considered three possible mechanisms that could explain why liposome encapsulation of uranine increases dye uptake by *C. elegans*, which are not mutually exclusive. (1) Liposomes retain the dye at the bacterial lawn surface making it more bioavailable. (2) Liposome encapsulation enables compounds to pass more easily across the intestinal cell surface, from the gut lumen, and into the body of the worm. (3) The particulate nature of the liposomes leads to more efficient transport by the pharynx into the intestine, as previously suggested [15, 16].

To test (1), 10 μL of 52.8 mM uranine, either free dye, or a mix of free and liposome-encapsulated dye, or purified, dye-loaded liposomes, was dropped onto the lawn surface, and the subsequent diffusion of the dye through the underlying agar measured at successive time intervals. As suspected, liposome encapsulation reduced dye diffusion into the agar (Fig. S3). Thus, liposome encapsulation causes compounds to remain concentrated at the lawn surface and prevents their diffusion into the agar.

If (2) is correct, then liposome encapsulation should allow any fluorescent dye to cross from the intestinal lumen into the interior of intestinal cells. To probe this, we tested a small selection of different fluorescent dyes: two that are more membrane permeable, uranine (fluorescein sodium salt) (MW 376.27) and acridine orange (MW 265.35), and one with poor membrane permeability, Texas red (sulforhodamine 101 acid chloride) (MW 625.16). Each dye was administered to worms with or without liposome encapsulation. To check for uptake both into the gut lumen and from there into the worm body, fluorescence was imaged either with or without a chase, i.e., incubation on dye-free *E. coli* after dye exposure to flush dye from the intestinal lumen.

**Fig. 2:**
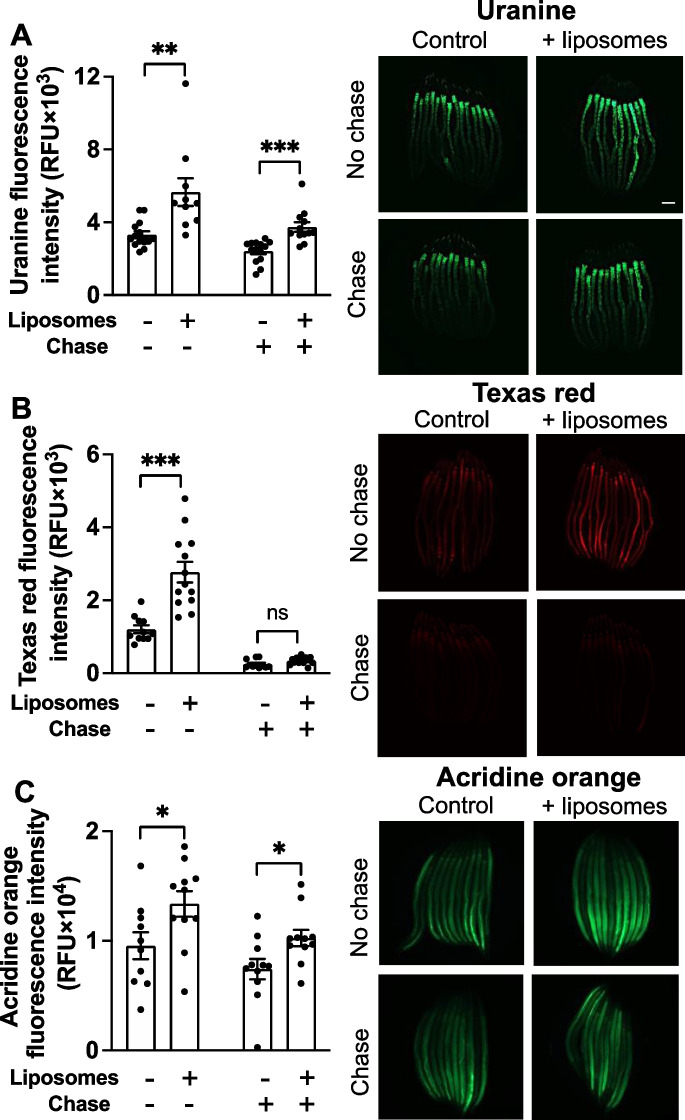
Liposomes increase Texas red ingestion but not uptake into the body. **A**, Uranine. **B**, Texas red. **C**, Acridine orange. Note that Texas red is only visible in the intestinal lumen, in worms without a chase. Scale bar, 100 μm. Mean ± S.E.M, *p < 0.05, **p < 0.01, ***p < 0.001, one-way ANOVA (Šidák correction)

Notably, while uranine and also acridine orange were detected in both the gut lumen and interior of intestinal cells (Figs. 2A, C and S4A, C), Texas red was detected only in the gut lumen (Figs. 2B and S4B). This implies that liposomes are not a means to ensure that any drug will cross the intestinal lumen. However, liposome encapsulation did increase the amount of Texas red in the gut lumen (Fig. 2B). This could be due to the retention of dye at the lawn surface and/or better transfer of dye by the pharynx into the gut lumen due to its particulate nature.

Liposome-encapsulated uranine crossed from the intestinal lumen, but liposome-encapsulated Texas red did not. We therefore wondered whether the inclusion of uranine in Texas red-containing liposomes might somehow enable the red dye to hitch a ride with the green across the apical membrane. However, when we tried this, only the uranine was taken up (data not shown).

**Fig. 3:**
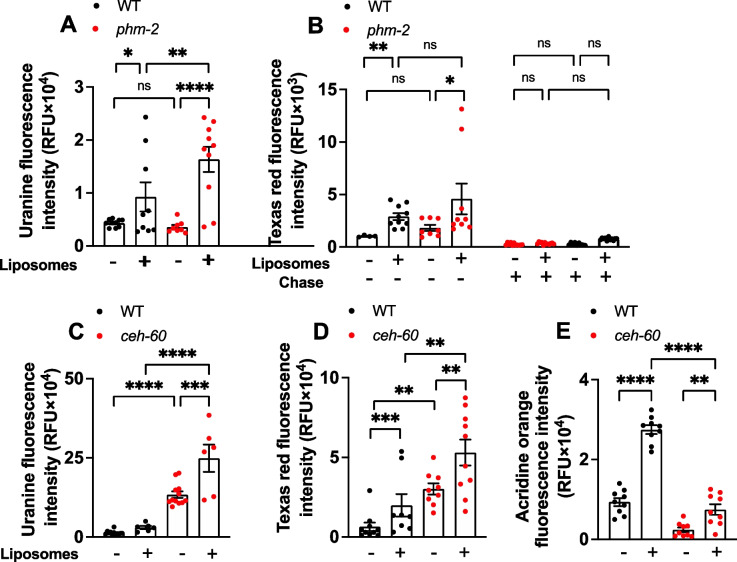
Liposome-mediated dye uptake by two *C. elegans* mutants. **A**, **B** *phm-2(ad597)* mutants with a grinder defect show increased dye uptake when delivered in liposomes with uranine (**A**), but not for Texas red (**B**). **C**, **D** *ceh-60(lst466)* mutants with increased cuticular permeability show increased uranine and Texas red uptake in liquid. **E** *ceh-60* mutants, surprisingly, show reduced AO uptake on plates. Mean ± S.E.M, *p < 0.05, **p < 0.01, ***p < 0.001, one-way ANOVA (Šidák correction)

Next, we attempted to further improve drug delivery by combining liposome encapsulation with the use of mutant *C. elegans* strains. One concern about liposome-mediated delivery is that liposomes might be destroyed by the grinder in the terminal bulb of the pharynx. An indication of the high degree of masticatory efficiency of this organ is that prior to adulthood, no bacteria make it through the grinder alive into the intestinal lumen [26]. *phm-2* mutants (Pharyngeal Muscle 2) have a defective pharynx in which, effectively, the jaws of the pharyngeal grinder are unable to close, allowing bacteria to pass into the lumen unmasticated [27, 28]. If the grinder does rupture liposomes passing through it, then mutation of *phm-2* should increase intestinal uptake of liposome-encapsulated uranine. In fact, this was the case: uranine uptake by *phm-2(ad597)* mutants was significantly higher than in the N2 wild type, but only when administered in liposomes (Fig. 3A). This suggests that the action of the grinder does reduce liposome-mediated dye uptake and that some liposomes are popped by the grinder. However, this effect was not seen when tested using Texas red (Fig. 3B), for unknown reasons.

It was previously shown that mutation of *ceh-60* (*C. elegans* homeobox) results in reduced vitellogenin production and increased cuticular permeability, demonstrable by a substantial increase in acridine orange (AO) uptake by worms incubated in a dye solution in buffer [29]. This suggests that *ceh-60* mutants might provide a means to ensure the uptake of any drugs by *C. elegans*. We first verified that dye uptake was increased in *ceh-60(lst466)* mutants in a liquid medium, and it was for uranine and Texas red (Fig. 3C, D) and also, as previously reported [29], for AO (data not shown). However, on NGM plates, *ceh-60* did not increase dye uptake, either with free or liposome-encapsulated dye for AO (Fig. 3E). Unexpectedly, dye uptake was lower in *ceh-60*, and more so when liposomes were used. This could imply either that liquid culture induces the hyperpermeability phenotype or that the freer movement of solutes in liquid is required for *ceh-60* cuticular hyperpermeability to be detectable using dye uptake assays.

It is notable that in buffer uptake of even Texas red was increased by *ceh-60* (Fig. 3D), pointing to the utility of this mutant to ensure uptake of even drugs with poor membrane permeability (in liquid medium, at least). Also, that liposome encapsulation increased dye uptake in buffer (in the absence of concentration of liposomes at the lawn surface) implies the presence of post-ingestion benefits, either due to their particulate nature aiding transport into the intestinal lumen or to fusion with the plasma membrane carrying contents into intestinal cells.

### Liposome-encapsulated glutathione extends lifespan by increasing infection resistance

Next, we tested the utility of liposome-mediated compound delivery for studies of drugs that extend *C. elegans* lifespan, i.e., that have potential anti-aging effects, using a range of compounds previously reported to have such effects. In these trials, unless otherwise stated, the same overall quantity of the drug was added to each plate, whether into the agar (in the control) or with liposome encapsulation (Table 1).

A previous study [16] found that four compounds increased lifespan only when liposome-encapsulated. These were three with potential antioxidant properties, *N*-acetylcysteine (NAC), vitamin C (ascorbate), and GSH, and an anticonvulsant, trimethadione, shown to increase *C. elegans* lifespan in an earlier study [30]. The effects of the antioxidants were somewhat surprising given that the once influential oxidative damage theory of aging was largely refuted over a decade ago [31-33]. We repeated these tests using the same compounds and were able to reproduce the previous findings for two of them, GSH and trimethadione (Supplemental Table 1). For all lifespan trials, statistical data is presented in Supplemental Tables, and raw data in the form of Ziehm tables (with full details of trial conditions) in Supplemental Dataset 1. Liposome encapsulation caused GSH to significantly increase lifespan in 4/4 trials, and for trimethadione in 2/2 trials; without liposomes, no effect on lifespan was seen with either compound. Trials with NAC (*N* = 3) and vitamin C (*N* = 2) gave largely negative results (Figure S5 and Supplemental Table 1).

**Fig. 4:**
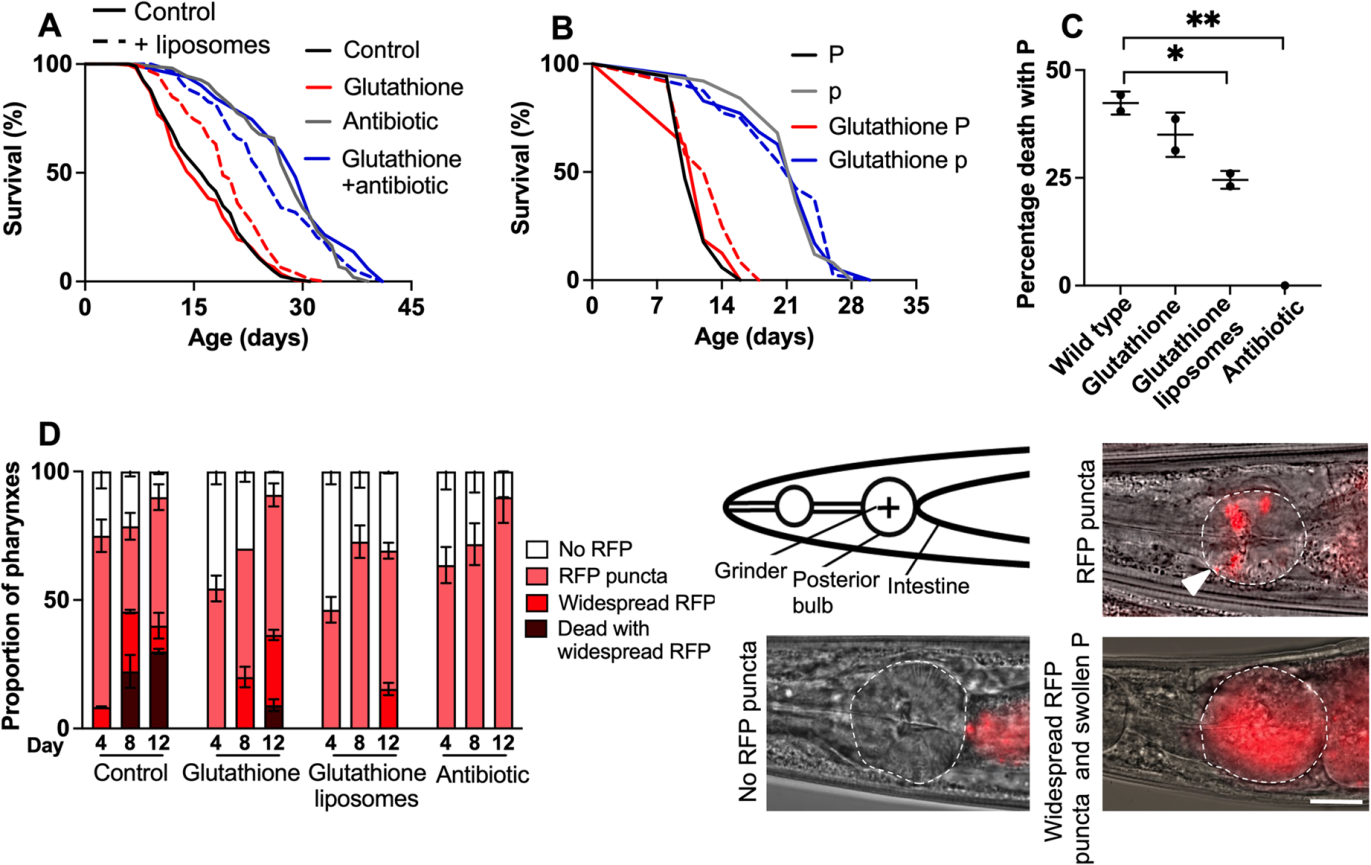
Effects of liposome-encapsulated GSH on *C. elegans* lifespan. **A** GSH does not increase lifespan in the absence of bacterial infection. **B** GSH has no effect on P or p lifespan. **C** GSH reduces the proportion of P deaths. Mean ± S.E.M., one-way ANOVA (Šidák correction). **B**, **C** together indicate that the increase in overall lifespan from GSH treatment is the result solely of reducing P death frequency. **D** Left: GSH reduces bacterial infection in the pharynx. Right: RFP-labeled *E. coli* in the *C. elegans* pharynx. Top right, red puncta (e.g., white arrow) shows sites of localized infection within the pharynx. Scale 50 μm. Mean ± S.E.M, *p<0.05, **p<0.01, one-way ANOVA (Šidák correction)

Having previously conducted a series of investigations of the oxidative damage theory that did not support it [34-38], we were curious about the life-extending effect of GSH, which is consistent with this theory. To explore this, we first tested the possibility that GSH acts by reducing the life-shortening effects of the bacterial food source. Elderly worms become susceptible to bacterial infection, and preventing this by blocking bacterial proliferation substantially increases *C. elegans* lifespan [2, 4]. If GSH were extending *C. elegans* lifespan by reducing accumulation of oxidative damage, it should do so in the absence of bacterial proliferation. In fact, treatment with the antibiotic carbenicillin abrogated life extension by GSH (Figs. 4A and S6; *N* = 4; Supplemental Table 1). Thus, the life-extending effect of GSH is unlikely to be due to the prevention of oxidative damage.

Suppression of GSH effects by carbenicillin suggests that GSH reduces bacterial infection in *C. elegans*. This could potentially be due to an antibiotic effect on the *E. coli*, reducing their pathogenicity, or to an increase in worm immunity to infection. That liposome encapsulation is expected to reduce exposure of *E. coli* to drugs is consistent with the latter interpretation; however, it is also possible that GSH taken up by the worms provides them with antibiotic defense. To explore this further, we first employed the technique of mortality deconvolution, which combines survival and necropsy analysis. During aging of wild-type *C. elegans* populations, ~ 40% of animals develop a fatal bacterial infection of the pharynx, which causes earlier death. Here, worm corpses have swollen pharynxes and, hence, are referred to as a P (“big P”) deaths. Later deaths produce corpses with atrophied pharynxes, referred to as p (“small p”) deaths [3].

Preventing bacterial infection abrogates P death and extends p lifespan [3]. Thus, if GSH had a mild antibiotic effect, one would expect a reduction in %P and an increase in p lifespan. In fact, GSH reduced %P without increasing p lifespan. Mortality deconvolution showed that the increase in overall (P + p) lifespan by GSH was solely attributable to reduced %P (Fig. 4B, C, *N* = 2; Supplemental Table 2).

P death appears to result from an early and a late event. In early adulthood, activity-dependent mechanical senescence of the pharyngeal cuticle allows small amounts of bacteria to cross from the pharyngeal lumen and into the pharyngeal muscle, where it is contained by host immunity. In later life, these latent infections recrudesce (i.e., re-emerge), and destroy the pharynx [3]. These two stages can be rendered visible by fluorescent labeling of *E. coli*, e.g., with RFP. GSH reduced both early infection and later recrudescence, consistent with a life-long increase in innate immunity (Fig. 4D).

Notably, it was recently shown that GSH induces features of trained immunity in human cells, via an unresolved mechanism [39]. Moreover, we previously showed that changes in mitochondrial morphology consistent with innate immune training occur in *C. elegans* treated with fumarate [40], which in mice can cause innate immune training [41]. Fumarate, like GSH, also reduces P death frequency but does not increase p lifespan [40].

**Fig. 5:**
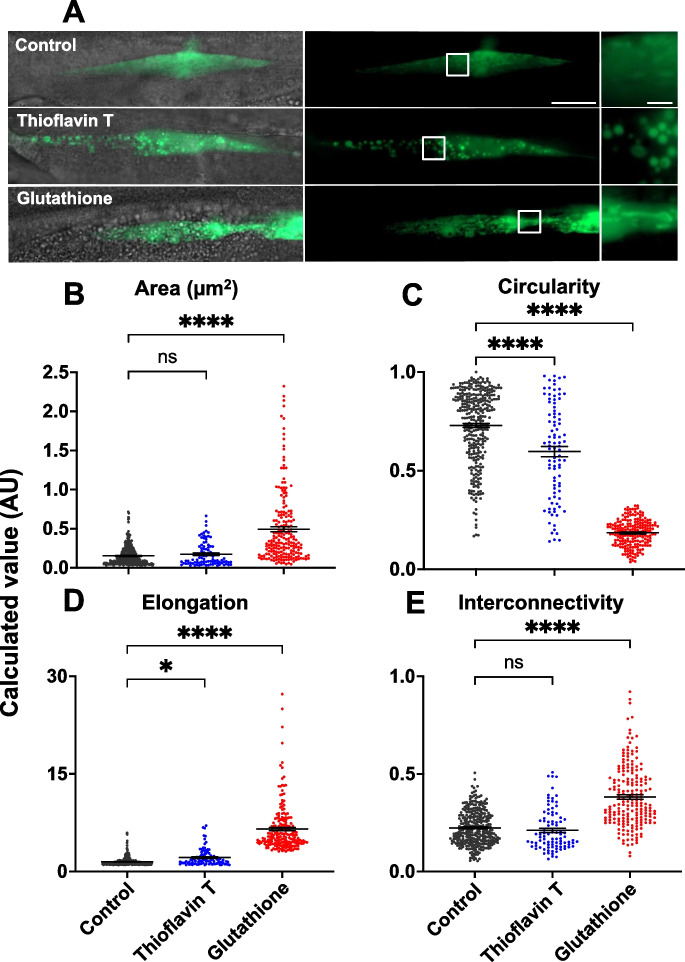
Evidence that GSH induces mitochondrial fusion in muscle cells of *C. elegans*. Images depict mitochondrial morphology in untreated (control) and GSH-treated *C. elegans*. **A** Mitochondrial morphology in strain SJ4103 *zcIs14*(P*myo-3*::GFP^mt^) (GFP-expressed in mitochondria of body wall muscle). Left: full muscle fiber, right: zoomed in section shown in box. Scale bar, left 20 µm, right 5 µm. **B**–**E** Muscle mitochondrial morphology parameters assessed by measuring all mitochondria within the image for treatment with ThT and GSH vs control (*N* = 15 animals). *p < 0.05, ****p < 0.0001; one-way ANOVA (Šidák correction). For intestinal mitochondria and images of ThT see supplemental Figure S7

To explore possible innate immune training properties of GSH in *C. elegans*, we looked at effects on mitochondrial morphology using a strain with GFP-labeled mitochondria in body wall muscle and intestinal cells. Animals were exposed to GSH at the L4 stage. Twenty-four hours later, mitochondria showed significantly reduced mitochondrial circularity as well as increased area, interconnectivity, and elongation, compared to untreated controls (Figs. 5A–E and S7). This evidence of mitochondrial fusion is consistent with an immune training effect (though it does not demonstrate it). We conclude that life-extending effects of GSH are unlikely to be due to protective effects against oxidative damage. Instead, GSH appears to increase host innate immunity, particularly to infection of the pharynx by *E. coli* in early adulthood.

### Life extension by thioflavin T is caused by its antibiotic properties

To further test the utility of liposome-mediated delivery for studies of putative anti-aging drugs, we tested two additional compounds previously reported to increase *C. elegans* lifespan: ThT and rapamycin. We also tested the dependency of drug effects on *E. coli* proliferative capacity.

**Fig. 6:**
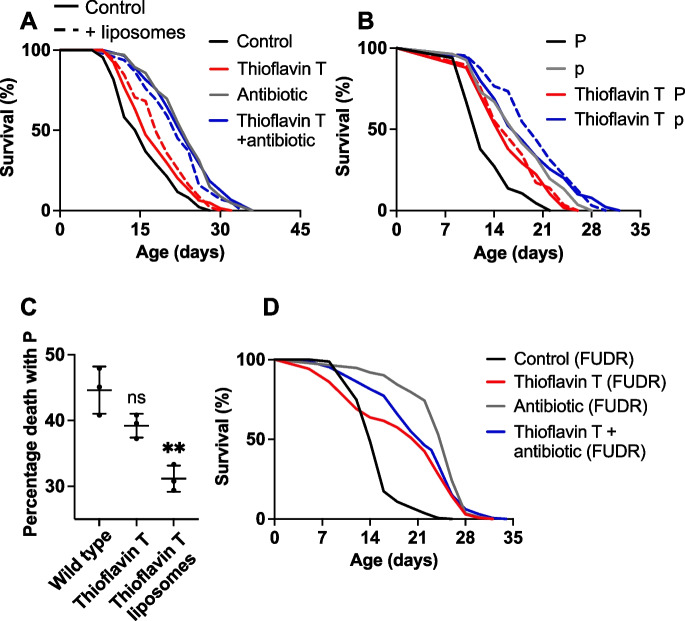
Effects of ThT on lifespan. **A** Effects of ThT on lifespan ± liposome encapsulation, and effects of ThT on lifespan are suppressed by carbenicillin. The concentration of ThT used (25 μM) was lower than in a prior study [42] (50 or 100 μM) in order to reduce frequency of death due to internal hatching. **B** ThT effects on P and p lifespan. **C** ThT effect on P death frequency. **D** Effect of 50 μM ThT on lifespan in the presence of FUDR is suppressed by carbenicillin. Mean ± S.E.M, **p < 0.01, one-way ANOVA (Šidák correction)

ThT is a yellow benzothiazole salt often used as a histological stain to identify amyloid, which increases *C. elegans* lifespan [42]. ThT increased lifespan whether liposome encapsulated or not, and liposome encapsulation slightly increased the magnitude of the lifespan increase. However, in the presence of carbenicillin ThT did not increase lifespan (Fig. 6A, S8; *N* = 3; Supplemental Table 3). The effect of ThT on lifespan, in the presence of bacterial proliferation, was due mainly to a reduction in P death frequency and an extension of P lifespan (Fig. 6B, C and Supplemental Table 3). Lifespan-extending effects of ThT reported previously were seen in the presence of 5-fluorodeoxyuridine (FUDR), used to block progeny production [42]. Suppression of this effect by carbenicillin was also seen in the presence of FUDR (Figs. 6D and S9).

The absence of an effect of ThT in the presence of carbenicillin suggests that life-extending effects are due to the prevention of bacterial infection; previous tests of ThT effects were performed in the presence of proliferating *E. coli* [42-44]. This could reflect either induction of immunity, as seen with GSH, or a simple antibiotic effect. To probe for possible induction of immunity, effects of ThT on mitochondrial morphology that could indicate an innate immune training effect were examined, but none were seen (Figs. 5 and S7).

**Fig. 7:**
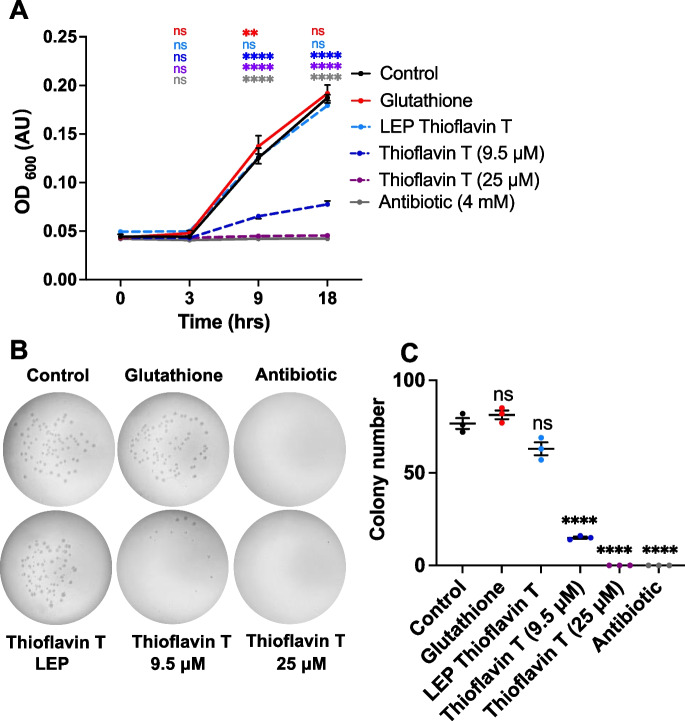
Effects of ThT on bacterial proliferation. **A** ThT suppresses *E. coli* OP50 growth in liquid culture. Mean ± S.E.M. Two-way ANOVA (Dunnett correction). **B**, **C** ThT suppresses bacterial growth on solid NGM. **B** Images of NGM plates. **C** Bacterial colony number (3 trials). Mean ± S.E.M., **p < 0.01, ****p < 0.0001, one-way ANOVA (Šidák correction)

By contrast, ThT suppressed bacterial growth, both in liquid culture (Fig. 7A) and when added to plates (Fig. 7B, C). This is in line with a recent observation that ThT can inhibit *E. coli* elongation rate, i.e., has an anti-proliferative effect [45]. Antibiotic effects were seen both at the concentration of free drug added to plates (25 μM) and at that resulting from free drug accompanying liposomes (estimated as ~9.5 μM; for the assay calibration curve, see Fig. S10). Taken together, these results suggest that ThT increases *C. elegans* lifespan in a manner similar to carbenicillin, by suppressing bacterial infection. However, an additional induction of immunity effect cannot be ruled out.

Notably, purified liposomes containing ThT did not reduce bacterial proliferation (Fig. 7A-C), demonstrating the capacity of liposome encapsulation to prevent drug-bacteria interactions. Moreover, GSH did not show any antibiotic effects (Fig. 7A–C), again suggesting that it acts on *C. elegans* to induce immunity rather than having a direct antibiotic effect.

### Bacterial proliferation can mask the effect of rapamycin on lifespan

Rapamycin is a macrolide compound that can inhibit the mammalian (or mechanistic) target of rapamycin (mTOR) kinase. It has been reported in several studies to increase lifespan in *C. elegans* [46-50]. Rapamycin proved to be somewhat challenging to work with due to its hydrophobicity. To maximize the quantity that could be delivered using liposomes required establishing the highest concentration of organic solvents (dimethylsulfoxide [DMSO] or ethanol) that could be used without causing liposome breakdown. This was assessed by measuring the relative increase in uranine uptake upon liposome encapsulation. This proved to be 5% for DMSO, while for ethanol, even the lowest concentration tested (2.5%) abrogated the liposome-mediated increase in dye uptake (unsurprisingly) (Fig. S11A).

Before testing the effects of rapamycin on lifespan, we first checked for possible effects of DMSO on survival. 5% DMSO had no significant effect on survival, either in the agar or delivered in liposomes, or with or without carbenicillin (Fig. S11B and Supplemental Table 5), though slight, non-significant trends towards reductions in lifespan were seen. This was also the case for empty liposomes (containing only water; Supplemental Table 5). We conclude that it is unlikely that any drug-induced increases in lifespan seen are due to either the 5% DMSO, or to the liposomes themselves.

**Fig. 8:**
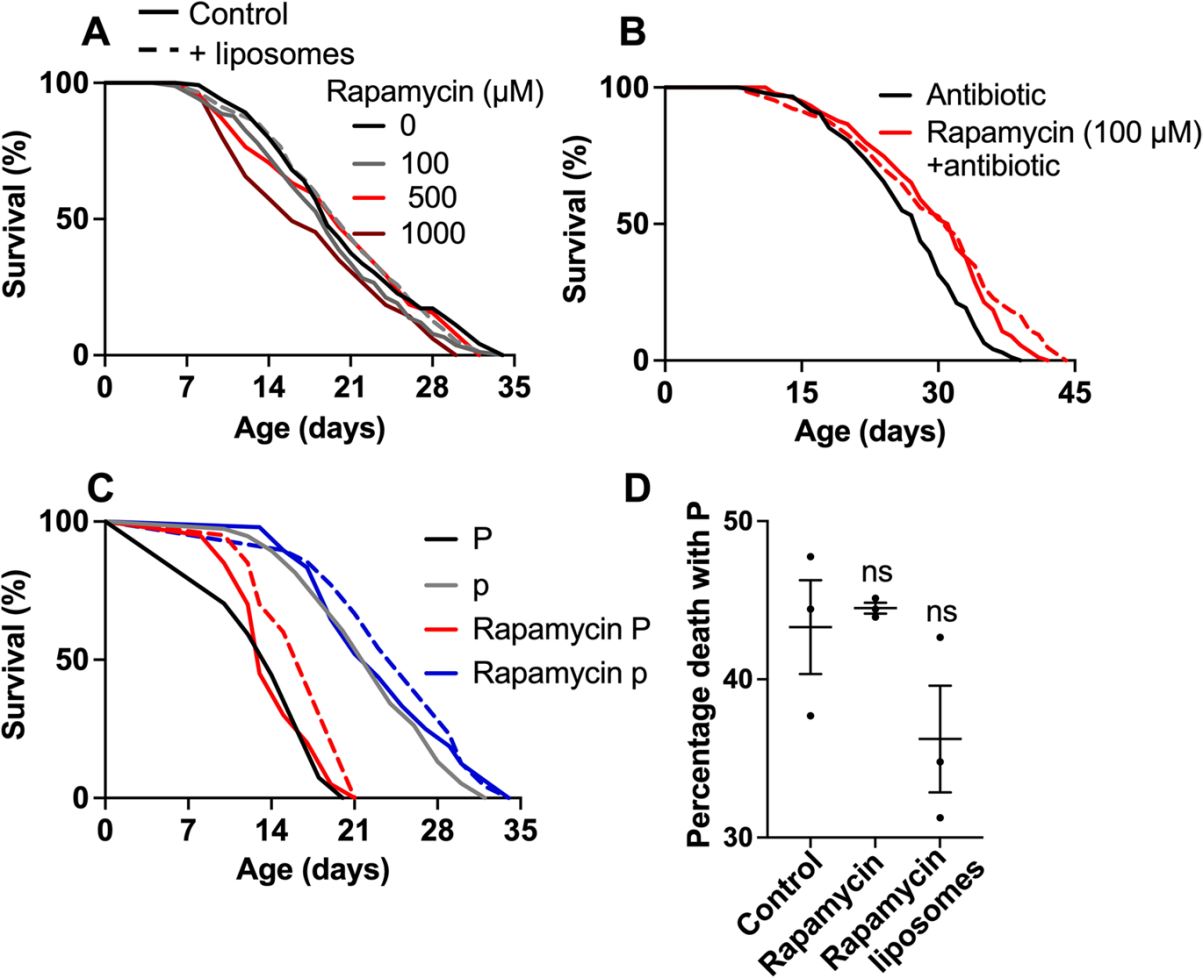
Effects of rapamycin on lifespan. **A** Little effect of rapamycin, with or without liposomes. **B** Rapamycin increases lifespan in the presence of antibiotic (carbenicillin). **C** Rapamycin causes an increase in p lifespan. **D** Rapamycin does not significantly reduce the proportion of P deaths, but when liposome encapsulated a non-significant trend towards a decline is seen. Mean ± S.E.M, one-way ANOVA (Šidák correction).

Rapamycin was added to molten agar to 100 μM, as previously described [46]; delivering the equivalent quantity of drug via liposomes was not feasible due to solubility limitations. In plates containing 100 μM rapamycin, some precipitation of the drug was observed, leading to an irregular agar surface, with a rocky appearance created by lumps of the precipitate. This effect has been observed by other investigators (Seung-Jae Lee, Alex Mendenhall, personal communication). No statistically significant increases in worm lifespan were seen with rapamycin in the agar (Figs. 8A and S12, *N* = 3), and with liposome-encapsulated drugs, a significant increase was seen in only 1 out of 3 trials, though non-significant trends towards increased lifespan were also seen under both conditions (Supplemental Table 6).

Next, the effects of rapamycin on lifespan on carbenicillin-treated plates were tested. Unexpectedly, inclusion of carbenicillin caused rapamycin in liposomes to robustly increase lifespan (Figs. 8B and S12, 3/3 trials; Supplemental Table 6). On proliferating *E. coli*, mortality deconvolution revealed slight increases in both P and p lifespan, and, when in liposomes, a slight trend for reduced P frequency (Fig. 8C, D and Supplemental Table 6). Thus, while the life-extending effects of GSH and ThT are dependent upon bacterial proliferation, those of rapamycin are suppressed by it. These results suggest that death from bacterial infection masks a senescence-related cause of mortality that is suppressed by rapamycin. That 50 μL of liposomes containing 100 μM rapamycin increases lifespan similarly to 100 μM in 10 ml of agar provides a means to reduce drug usage by 200-fold (which is useful given the high cost of this drug).

### No improvement of growth on axenic medium in extruder-generated liposomes

A mysterious feature of *C. elegans* is that it will only grow in the presence of metabolically active microbes [5, 51]. No axenic medium, defined or semi-defined has been identified on which it will grow and reproduce normally. However, it was reported several years ago that if axenic medium is provided encapsulated in liposomes, *C. elegans* will grow and reproduce normally upon it [15]. The authors suggested that this could be because *C. elegans* require food in particulate form to be consumed. This could provide a means to study *C. elegans* aging in the absence of *E. coli* and avoid confounding *E. coli*-mediated effects of drugs, which would be very useful.

To explore this possibility, we compared growth of *C. elegans* larvae with free or liposome-encapsulated axenic medium, with liposomes prepared using the liposome extruder. However, no improvements to growth were seen (Fig. S13A). The differences between our results and those of the previous study may reflect the use of different protocols for liposome generation. In the earlier study, vortexing was used to prepare liposomes, thereby producing liposomes of mixed sizes with different diameters. We tried using a mix of extruder-generated liposomes of different sizes (generated with different filters) but without success (Fig. S13B).

## Discussion

In nematodes, liposomes have been previously used for delivery of drugs [16, 25] and antibodies [52] and RNAi and CRISPR-Cas9 genome editing [53]. In this study, we provide further demonstration of the utility of liposomes for studies of the effects of drugs on aging in *C. elegans*, and details of their mode of action. In particular, we show how drug effects on *C. elegans* can be altered by both liposome encapsulation and bacterial status. These findings should help with design of future studies of drug effects on nematode aging, including high-throughput screens [1], whether in the context of scientific research [44, 54, 55], or commercial drug screening services such as those offered by Ora Biomedical in the USA, using the WormBot robotic system [56], and Magnitude Biosciences in the UK [57]. Practically speaking, this could involve robotic production and/or administration of liposomes, if that were feasible. Failing that, liposome-mediated delivery could be used to manually assess effects of life-extending compounds identified using automated screens. The former would be preferable in principle, since it would improve life-extending drug identification.

Our findings confirm those of an earlier study [16], that liposome encapsulation increases compound uptake by *C. elegans*. However, how exactly it does had remained unclear. Our findings imply that a major mechanism is increased compound bioavailability due to concentration of liposomes on the bacterial lawn surface. The possibility that it is the particulate nature of liposomes that improves uptake by *C. elegans* is less certain; improved uptake of dyes by liposome encapsulation in liquid culture (Fig. 3) is consistent with such a mechanism. Our hope was that liposomes might provide efficient passage into nematodes for any drug, including membrane-impermeable ones, but the lack of uptake of Texas red argues against this.

These studies also underscore that drugs that extend the lifespan in *C. elegans* may do so by reducing bacterial infection. Of the three life-extending compounds studied in detail, the effects of one, rapamycin, proved to be independent of bacterial effects. By contrast, GSH and ThT appear to act by reducing bacterial infection; neither increased lifespan when bacterial infection was blocked using an antibiotic.

In the case of GSH, liposome-encapsulated GSH reduced P death without increasing p lifespan, suggesting an enhancement of immunity in the pharynx. One possibility is that this reflects enhancement of innate immunity. We recently reported a similar effect from treatment with fumarate (not in liposomes), which is thought to be the result of innate immune training [40]. The effects of GSH on mitochondrial morphology (Fig. 5) and its known immune training effect (in mice) [39] are consistent with this view. Whether or not this hypothesis is correct, GSH treatment effects could involve the complex *C. elegans* thiol network [58], including GSH-dependent enzymes such as glutathione peroxidases and glutathione S-transferases.

By contrast, ThT did not affect mitochondrial morphology (Fig. 5) but, in contrast to GSH, did exhibit antibiotic properties (Fig. 7), consistent with several recent bacteriological studies [45, 59]. This implies that ThT, like carbenicillin, increases *C. elegans* lifespan by preventing life-shortening bacterial infection rather than, as previously suggested [42], by alleviating protein aggregation. Previous tests of effects of ThT on *C. elegans* lifespan were performed in the presence of live *E. coli* [42-44, 60]. An alternative explanation (less parsimonious) is that ThT can increase lifespan by reducing protein aggregation, but only when proliferating *E. coli* are present. If this were the case, one might expect that the antibiotic effects of ThT would additionally extend lifespan by reducing infection.

In the case of rapamycin, robust increases in lifespan were only seen when bacterial proliferation was prevented, using an antibiotic (Fig. 8). This is to some extent consistent with earlier studies. One study using UV-killed *E. coli* saw increased lifespan with as little as 10 μM rapamycin [49], and another using 100 μM rapamycin added ampicillin to plates [50]. However, other studies used live *E. coli* and 100 μM rapamycin [46], or at least an ampicillin-resistant L4440 RNAi control strain with antibiotic present [47, 48]. While ampicillin resistance assures normal proliferation of *E. coli* in the presence of carbenicillin (Fig. S14A), the IPTG (isopropyl β-d-1-thiogalactopyranoside) included in plates to induce synthesis of target gene RNA does inhibit *E. coli* proliferation (Fig. S14B). Thus, it is possible that the IPTG in RNAi tests potentiates life-extending effects of rapamycin in *C. elegans* by reducing bacterial infection. Alternatively, other inter-laboratory differences in culture conditions could be operative here, and possibly non-publication of negative findings (publication bias).

The behavior of rapamycin illustrates how life-extending effects may be masked by bacterial infection. One possibility is that this is due to an onion effect: where a life-limiting senescent pathology masks the effect on survival of amelioration of another senescent pathology. In other words, when infection is prevented, other pathologies become life limiting that can be suppressed by rapamycin (c.f. the layers of an onion) [61]. We are currently investigating the possible nature of such pathologies. Notably, the increase in *Drosophila melanogaster* lifespan induced by rapamycin is as great (if not greater) when microbial proliferation is inhibited [62].

The role of bacterial infection in drug effects on survival raises a question about experimental design: Are bacterially mediated effects informative with respect to mechanisms of aging, or would it be better to exclude them altogether by performing all trials in the absence of bacterial infection? We have previously argued for the former view [3]. For example, P death provides a model for understanding the relationship between extrinsic and intrinsic causes of aging; it makes possible mortality deconvolution, an approach to improve understanding of the relationship between biological and demographic parameters, and in the case of rapamycin, it points to the presence of ranked sequences of life-limiting pathologies (the onion model) [61]. Thus, what is important is not so much to exclude bacterially mediated effects on aging, as to understand them. Increased susceptibility to infection is an important feature of aging, as the COVID-19 epidemic demonstrated: probability of developing fatal severe acute respiratory syndrome after infection with SARS-CoV-2 virus increases exponentially with age.

In conclusion, liposome-mediated delivery provides a number of benefits, including reduction of drug quantity needed for efficient delivery into the worm (which reduces cost), reduction of drug biotransformation by bacteria, and reduction of drug effects on bacteria, including antibiotic effects. However, they do not solve all problems of drug uptake by *C. elegans*. What would help is a mutant strain that is both healthy and normal lived, but more easily takes up compounds. This has been explored previously by looking for strains with greater cuticular permeability, such as the *bus-5(br19)* (Bacterially UnSwollen 5) mutant [63], and, recently, the *gmap-1(ulb13)* (GM2 Activator Protein 1) mutant [64]. Perhaps better would be a strain with greater permeability in the intestine, if one could be isolated that did not reduce viability.

Finally, it is worth emphasizing that for most drugs examined in this study, only one concentration was tested. For liposome-mediated delivery, optimal concentrations for life extension need to be defined. Moreover, where lifespan effects were not seen, this may in some instances be due to the use of too low a concentration.

## Supplementary Information

Below is the link to the electronic supplementary material.

Supplementary file1 (DOCX 5779 KB)

Supplementary file2 (XLSX 278 KB)

## Data Availability

The online version of this article contains supplementary material, which is available to authorized users.
