# Supplementary material for: Unraveling effects of anti-aging drugs on C. elegans using liposomes
Source: GeroScience. 2023 May 4;45(3):1583–603. doi: 10.1007/s11357-023-00800-x (PMC10158714; doi:10.1007/s11357-023-00800-x)
Supplement: Supplementary file 1 — Supplementary file1 (DOCX 5779 KB) [file 11357_2023_800_MOESM1_ESM.docx]

**Supplemental Figures**

**Figure S1**. Verification of liposome formation. A, Reduced absorbance of lipid mixture after extrusion, corresponding to liposome formation. B, Use of liposome extruder purification (LEP) to confirm presence of liposomes. Here liposomes are placed in a syringe attached to a filter with pores too small for them to pass through (50 nm), but through which free dye can pass. The peak at 495 nm corresponds to liposomes loaded with uranine trapped by the 50 nm filter. C, TEM image of a liposome. Scale bar, 100 nm.

**Figure S2.** Three separate trials of serial exposure of dyes encapsulated in liposomes showing level of dye uptake is not likely due to intrinsic differences between worms. A weak significant positive correlation is seen with R^2^ > 0.26 in all trials, suggesting only around 20% of the variation in the data is explained by differences between individual worms.

**Figure S3**. Liposome encapsulation leads to dye retention at the plate surface. A, Experimental design. B, Images of green uranine fluorescence in agar slices at intervals after addition of dye to plate surface. C, Quantitation of decline in fluorescence with distance from point of application of dye to plate surface. Liposomes are a suspension of liposomes containing dye in a dye solution. LEP liposomes are purified liposomes, i.e. with dye within the liposomes but not the surrounding buffer. The latter show the least diffusion through the agar.

**Figure S4.** Distribution of different dyes in the intestine of wild-type *C. elegans*, zoomed in section shown in box. A, Uranine. B, Texas red. C, Acridine orange. Scale bar, left 100 µm, right 50 µm.

**Figure S5.** Three separate trials of effects of two antioxidants and trimethadione on *C. elegans* lifespan (20˚C). NAC (*N*=3) showed largely negative effect on lifespan, VC (*N*=2) showed weak effect in 1/2 trails, and trimethadione (*N*=2) only showed lifespan increase when liposomes were present. For statistical details, see Supplemental Table 1.

**Figure S6.** Four separate trials of effects of GSH on *C. elegans* lifespan (20˚C). GSH showed no lifespan increase in 4/4 trails, but 3/4 showed significant lifespan increase with using liposomes. For statistical details, see Supplemental Table 1

**Figure S7.** Evidence that GSH induces mitochondrial fusion in intestinal cells of *C. elegans* but ThT does not. Images depict mitochondrial morphology in untreated (control) and ThT- or GSH-treated *C. elegans*. A, Mitochondrial morphology in strain SJ4143 *zcIs17*(P*ges -1*::GFP^mt^) (GFP-expressed in mitochondria in intestinal cells). B-E, Intestinal mitochondrial morphology parameters assessed by measuring all mitochondria within the image. Scale bar, 10 µm. ****p* < 0.001, *****p* < 0.0001; one-way ANOVA (Šidák correction).

**Figure S8.** Effects of ThT (25 μM) on *C. elegans* lifespan (20˚C). ThT significantly increased lifespan in 3/3 trails, but the effect abrogated by carbenicillin. For statistical details, see Supplemental Table 3.

**Figure S9.** Effects of ThT (50 μM) on *C. elegans* lifespan (20˚C) with FUDR. ThT significantly increased lifespan in 3/3 trails, but the effect was abrogated by carbenicillin. For statistical details, see Supplemental Table 3.

**Figure S10.** ThT calibration curve. ThT (25 μM) solution in MilliQ water was diluted 1/2, 1/4, 1/8, 1/16, and 1/32, and the absorbance of each dilution measured using spectroscopy (ThT λ_ex_/λ_em_ 349 nm/454 nm), with a calibration curve derived via linear regression. R^2^= 0.99; *p* < 0.0001.

**Figure S11.** Effects of DMSO and ethanol. A, Effects on concentration on liposome-mediated delivery of uranine to *C. elegans*. Mean ± S.E.M., by one-way ANOVA (Šidák correction). B, Effects of DMSO on lifespan, and effects on that of liposome encapsulation, and antibiotics (carbenicillin). For statistical details, see Supplemental Table 4.

**Figure S12.** Effects of rapamycin on *C. elegans* lifespan (20˚C). Free rapamycin alone (100 μM) had little effect on lifespan in 3/3 trails, and liposome-encapsulated alone increased lifespan significantly in only 1/3 trails. With carbenicillin, rapamycin (free or liposome encapsulated) increased lifespan in 3/3 trails; Cox proportional hazard analysis, *p* < 0.0001. For statistical details, see Supplemental Table 5.

**Figure S13**. No enhancement of larval growth in axenic medium after liposome encapsulation. A, Liposomes of 500 nm diameter. B, Liposomes of different diameters. Mean ± S.E.M., by one-way ANOVA (Šidák correction). ***p* > 0.01.

**Figure S14**. *E. coli* HT115 growth in liquid culture is inhibited by IPTG. A, The ampicillin resistance gene on the RNAi plasmid assures normal growth in the presence of carbenicillin. B, IPTG suppresses *E. coli* growth. If bacterial proliferation masks life-extending effects of rapamycin on *C. elegans*, then it is possible that IPTG used in RNAi experiments promotes life extension by rapamycin.

**Supplementary Table 1**. Tests of effects of four compounds on lifespan, with or without liposome

encapsulation. C is combined data from all trials.

| **Strain/ condition** (compound concentration/plate) | **Number of deaths/ censored** | **All deaths** | | | | |
| --- | --- | --- | --- | --- | --- | --- |
|  |  | **Mean lifespan (days)** | **% change vs. control** | **p vs. control (log rank)** | **% change**  **vs. no liposomes used** | **p vs. no liposomes used**  **(log rank)** |
| Control (no drug, no liposomes) | [**C] 208/41**  [1] 64/5  [2] 51/9  [3] 53/7  [4] 40/20 | **16.75**  15.90  16.73  17.34  17.41 |  |  |  |  |
| GSH 117 µM | **[C] 203/48**  [1] 58/6  [2] 60/12  [3] 50/10  [4] 35/20 | **16.05**  14.51  16.84  16.46  16.82 | **-4.18**  -8.74  +0.66  -5.07  -3.39 | **0.42**  0.19  0.67  0.79  0.72 |  |  |
| GSH, liposomes, 117 µM | **[C] 203/39**  [1] 53/10  [2] 54/6  [3] 47/13  [4] 49/10 | **19.75**  18.09  18.39  20.96  21.96 | **+17.91**  +13.77  +9.92  +20.88  +26.13 | **<0.0001**  0.048  0.21  0.0002  0.011 | **+23.05**  +24.67  +9.20  +27.34  +30.56 | **<0.0001**  0.0008  0.53  0.0002  0.0059 |
| NAC, 12.5 µM | **[C] 164/26**  [1] 60/10  [2] 49/11  [3] 55/5 | **16.20**  14.54  16.39  17.91 | **-3.28**  -8.55  -2.03  +3.29 | **0.081**  0.098  0.57  0.63 |  |  |
| NAC, liposomes, 12.5 µM | **[C] 175/26**  [1] 68/4  [2] 57/12  [3] 55/10 | **17.34**  16.49  17.66  18.04 | **+3.52**  +3.71  +5.56  +4.04 | **0.38**  0.57  0.24  0.90 | **+7.04**  +13.41  +7.75  +0.73 | **0.032**  0.020  0.17  0.55 |
| Trimethadione, 838.5 µM | **[C] 90/26**  [1] 39/17  [2] 51/9 | **16.67**  16.87  16.53 | **-0.48**  +6.10  -1.19 | **0.48**  0.24  0.70 |  |  |
| Trimethadione, liposomes, 838.5 µM | **[C] 95/19**  [1] 49/11  [2] 46/8 | **19.90**  20.04  19.74 | **+18.81**  +26.04  +17.99 | **<0.0001**  <0.0001  0.0031 | **+19.38**  +18.79  +19.42 | **0.014**  0.067  0.085 |
| Vitamin C, 136.5 µM | **[C] 82/38**  [1] 38/22  [2] 44/16 | **16.13**  16.27  15.97 | **-3.70**  +2.33  +4.54 | **0.044**  0.047  0.14 |  |  |
| Vitamin C, liposomes, 136.5 µM | **[C] 104/18**  [1] 49/10  [2] 55/8 | **16.28**  16.50  16.07 | **-2.81**  +3.77  -3.95 | **0.060**  0.0005  0.016 | **+0.93**  +1.41  +0.63 | **0.22**  0.31  0.32 |
| Control  (carbenicillin 4 mM) | **[C] 210/33**  [1] 52/12  [2] 52/8  [3] 50/8  [4] 56/5 | **27.38**  28.13  26.63  29.80  25.71 |  |  |  |  |
| GSH 117 µM  (carbenicillin 4 mM) | **[C] 213/28**  [1] 51/9  [2] 51/9  [3] 54/5  [4] 57/5 | **28.69**  28.96  28.41  27.03  25.20 | **+4.78**  +2.95  +6.68  -9.28  -1.98 | **0.034**  0.14  0.10  0.0032  0.93 |  |  |
| GSH, liposomes, 117 µM  (carbenicillin 4 mM) | **[C] 199/38**  [1] 42/12  [2] 48/12  [3] 45/7  [4] 64/7 | **25.52**  25.76  25.35  25.79  25.76 | **-6.79**  -8.43  -4.84  -9.28  -1.98 | **0.37**  0.69  0.67  0.0032  0.93 | **-11.05**  -11.05  -10.77  -4.59  +2.22 | **0.0052**  0.044  0.071  0.32  0.87 |
| NAC 12.5 µM  (carbenicillin 4 mM) | **[C] 105/19**  [1] 48/12  [2] 57/7 | **27.17**  26.92  27.35 | **-0.77**  -4.30  +2.70 | **0.54**  0.92  0.77 |  |  |
| NAC, liposomes 12.5 µM  (carbenicillin 4 mM) | **[C] 105/17**  [1] 54/8  [2] 51/9 | **23.00**  23.23  22.82 | **-16.00**  -17.42  -14.31 | **<0.0001**  0.0086  0.0041 | **-15.35**  -13.71  -16.56 | **0.0003**  0.034  0.0014 |

**Supplementary Table 2**. Deconvolved effects of glutathione on mortality. C is combined data from all trials.

| **Strain/ condition** (compound conc/plate) | **Number of deaths/ censored** | **All deaths** | | | **P deaths** | | | | **p deaths** | | |
| --- | --- | --- | --- | --- | --- | --- | --- | --- | --- | --- | --- |
|  |  | **Mean lifespan (days)** | **% change vs. control** | ***p* vs. control (log rank)** | **Number/%P of death** | **Mean lifespan (days)** | **% change vs. control** | ***p* vs. control**  **(log rank)** | **Mean lifespan (days)** | **% change vs. control** | ***p* vs. control**  **(log rank)** |
| Control | **[C] 93/17**  [1] 52/6  [2] 41/11 | **17.73**  18.05  17.36 |  |  | **40/43.01**  23/44.23  17/41.46 | **11.65**  11.91  11.29 |  |  | **21.93**  22.41  21.36 |  |  |
| GSH 117 µM | **[C] 95/12**  [1] 44/3  [2] 51/9 | **17.76**  17.31  18.10 | **+0.06**  -4.10  +4.26 | **0.91**  0.65  0.36 | **33/34.74** 17/38.64  16/31.37 | **11.27**  10.71  11.88 | **-3.26**  -10.00  +5.26 | **0.59**  0.26  0.39 | **20.97**  21.41  20.63 | **-4.37**  -4.46  -3.42 | **0.63**  0.64  0.94 |
| GSH, liposomes, 117 µM | **[C] 103/16**  [1] 51/6  [2] 52/10 | **19.65**  20.09  19.20 | **+10.83**  +15.79  +10.60 | **0.023**  0.13  0.036 | **25/24.27**  13/25.49  12/23.08 | **12.80**  12.92  12.67 | **+9.87**  +8.48  +12.22 | **0.18**  0.54  0.11 | **21.56**  22.46  20.70 | **-1.68**  -0.03  -3.09 | **0.49**  0.53  0.50 |

**Supplementary Table 3**. Mortality deconvolution analysis of effects of ThT. C is combined data from all trials.

| **Strain/ condition** (compound concentration/plate) | **Number of deaths/ censored** | **All deaths** | | | **P deaths** | | | | **p deaths** | | |
| --- | --- | --- | --- | --- | --- | --- | --- | --- | --- | --- | --- |
|  |  | **Mean lifespan (days)** | **% change vs. control** | ***p* vs. control (log rank)** | **Number/%P of death** | **Mean lifespan (days)** | **% change vs. control** | ***p* vs. control**  **(log rank)** | **Mean lifespan (days)** | **% change vs. control** | ***p* vs. control**  **(log rank)** |
| Control | **[C] 148/32**  [1] 49/10  [2] 48/12  [3] 51/10 | **15.89**  15.54  15.55  16.57 |  |  | **66/44.59**  20/40.82  23/47.92  23/45.10 | **13.09**  12.40  12.78  14.00 |  |  | **17.88**  17.59  17.36  18.64 |  |  |
| ThT 25 µM | **[C] 148/115**  [1] 48/40  [2] 49/37  [3] 51/38 | **17.88**  18.16  17.62  17.87 | **+12.52**  +16.86  +13.31  +7.84 | **0.0023**  0.019  0.099  0.20 | **58/39.19**  19/39.58  20/40.82  19/37.25 | **16.28**  16.32  15.70  17.87 | **+24.37**  +31.61  +22.85  +26.64 | **<0.0001**  0.0064  0.011  0.038 | **18.76**  18.97  18.90  18.44 | **+4.92**  +7.85  +8.87  -1.07 | **0.17**  0.22  0.36  0.80 |
| ThT, liposomes, 25 µM | **[C] 93/111**  [1] 26/35  [2] 34/32 [3] 33/44 | **19.10**  19.25  19.19  18.85 | **+20.20**  +23.87  +23.41  +13.76 | **<0.0001**  0.0080  0.0078  0.068 | **29/31.18**  8/30.78  10/29.41  11/33.33 | **16.90**  17.50  15.80  18.85 | **+29.11**  +41.13  +23.63  +34.64 | **0.0002**  0.011  0.027  0.046 | **20.00**  19.89  20.50  19.55 | **+11.86**  +13.08  +18.09  +4.88 | **0.020**  0.17  0.054  0.37 |
| Control  (carbenicillin 4 mM) | **[C] 169/9**  [1] 56/4  [2] 56/3  [3] 57/2 | **23.53**  23.61  23.50  23.47 |  |  |  |  |  |  |  |  |  |
| ThT 25 µM  (carbenicillin 4 mM) | **[C] 119/108**  [1] 37/43  [2] 38/37  [3] 44/28 | **23.41**  23.46  22.53  22.05 | **-0.51**  -0.64  -4.12  -6.05 | **0.88**  0.77  0.68  0.19 |  |  |  |  |  |  |  |
| ThT, liposomes, 25 µM  (carbenicillin 4 mM) | **[C] 113/129**  [1] 35/47  [2] 34/40  [3] 44/42 | **22.14**  23.37  20.71  22.27 | **-5.91**  -1.02  -11.87  -5.11 | **0.056**  0.88  0.056  0.21 |  |  |  |  |  |  |  |

| **Strain/ condition** | **Number of deaths/ censored** | **All death** | | |
| --- | --- | --- | --- | --- |
|  |  | **Mean lifespan (days)** | **% change vs. control** | ***p* vs. control (log rank)** |
| Control  FUDR 75 μM | **[C] 167/11**  [1] 60/4  [2] 51/3  [3] 56/4 | **15.21**  15.70  14.79  15.08 |  |  |
| ThT 50 μM + FUDR 75 μM | **[C] 144/14**  [1] 50/4  [2] 55/3  [3] 39/7 | **19.09**  17.37  19.18  21.05 | **+25.51**  +10.64  +29.68  +39.59 | **<0.0001**  0.0033  <0.0001  <0.0001 |
| Control  FUDR 75 μM  (carbenicillin 4 mM) | **[C] 170/4**  [1] 54/1  [2] 60/3  [3] 56/0 | **23.94**  24.17  23.80  23.89 |  |  |
| ThT 50 μM + FUDR 75 μM  (carbenicillin 4 mM) | [C] **166/8**  [1] 57/4  [2] 56/2  [3] 53/2 | **21.18**  22.24  20.96  20.26 | **-11.52**  -7.99  -11.92  -15.19 | **0.0007**  0.23  0.025  0.014 |

**Supplemental Table 4**. Effects of Thioflavin on lifespan with FUDR. C is combined data from all trials.

| **Strain/ condition** (compound concentration/  plate) | **Number of deaths/ censored** | **All deaths** | | |
| --- | --- | --- | --- | --- |
|  |  | **Mean lifespan (days)** | **% change vs. control** | ***p* vs. control (log rank)** |
| Control | **[C] 117/12**  [1] 55/6  [2] 62/6 | **16.81**  16.67  18.48 |  |  |
| Lipid | **[C] 115/12**  [1] 54/9  [2] 61/3 | **16.31**  17.11  16.28 | **-2.97**  +2.64  -13.56 | **0.31**  0.07  0.45 |
| Liposomes | **[C] 108/12**  [1] 55/5  [2] 53/7 | **16.11**  16.87  15.28 | **-4.16**  +1.20  -17.31 | **0.06**  0.18  0.05 |
| 5% DMSO | **[C] 103/11**  [1] 45/5  [2] 58/6 | **15.89**  15.22  17.71 | **-5.47**  -8.70  -4.17 | **0.08**  0.33  0.45 |
| 5% DMSO  liposomes | **[C] 118/18**  [1] 57/12  [2] 61/6 | **16.33**  19.13  16.65 | **-2.86**  +14.76  -9.90 | **0.09**  0.39  0.78 |
| Control  (carbenicillin 4 mM) | **[C] 115/12**  [1] 52/8  [2] 63/4 | **27.68**  27.91  26.63 |  |  |
| Liposomes  (carbenicillin 4 mM) | **[C] 111/20**  [1] 52/10  [2] 59/10 | **27.15**  26.88  27.65 | **-1.91**  -3.69  +3.83 | **0.09**  0.60  0.50 |
| 5% DMSO  (carbenicillin 4 mM) | **[C] 101/14**  [1] 46/9  [2] 55/5 | **27.45**  28.55  26.71 | **-0.83**  +2.29  +0.30 | **0.05**  0.73  0.97 |
| 5% DMSO  liposomes  (carbenicillin 4 mM) | **[C] 94//16**  [1] 53/7  [2] 41/9 | **25.10**  24.08  26.98 | **-9.32**  -13.72  +1.31 | **0.06**  0.10  0.85 |

**Supplementary Table 5**. Effects of DMSO on lifespan. C is combined data from all trials.

**Supplementary Table 6**. Effects of different doses of rapamycin, and ± antibiotics on lifespan. C, combined data from all trials.

| **Strain/ condition** (compound concentration/plate) | **Number of deaths/ censored** | **All deaths** | | | **P deaths** | | | | **p deaths** | | |  |
| --- | --- | --- | --- | --- | --- | --- | --- | --- | --- | --- | --- | --- |
|  |  | **Mean lifespan (days)** | **% change vs. control** | ***p* vs. control (log rank)** | **Number/%P of death** | **Mean lifespan (days)** | **% change vs. control** | ***p* vs. control**  **(log rank)** | **Mean lifespan (days)** | **% change vs. control** | ***p* vs. control**  **(log rank)** | **Cox PH Prob>Chisq** |
| Control | **[C] 151/21**  [1] 52/7  [2] 35/9  [3] 64/5 | **17.90**  18.26  17.44  15.90 |  |  | **65/43.05**  20/38.46  17/48.57  28/43.75 | **15.71**  14.87  16.13  15.93 |  |  | **22.70**  22.47  23.03  22.63 |  |  |  |
| Rapamycin  100 µM | **[C] 176/21**  [1] 65/4  [2] 45/10  [3] 66/7 | **18.99**  20.30  17.42  16.79 | **+6.09**  +11.17  -0.11  +5.60 | **0.077**  0.080  0.86  0.54 | **78/44.32**  28/43.07  20/44.44  30/45.45 | **16.34**  16.55  16.75  15.76 | **+4.01**  +11.30  +3.84  -1.07 | **0.26**  0.036  0.49  0.81 | **23.09**  23.40  23.75  22.10 | **+1.72**  +4.14  +3.13  -2.34 | **0.35**  0.40  0.61  0.86 |  |
| Rapamycin  0.5 µM  liposomes | **[C] 159/22**  [1] 45/7  [2] 46/11  [3] 68/4 | **18.43**  18.45  19.19  18.10 | **+2.96**  +1.04  +10.03  +13.84 | **0.056**  0.61  0.34  0.029 | **59/37.11**  14/31.11  16/34.78  29/42.64 | **16.70**  16.55  16.75  16.75 | **+6.30**  +11.30  +3.84  +5.14 | **0.18**  0.047  0.79  0.36 | **24.78**  24.88  24.76  25.33 | **+9.16**  +10.73  +7.51  +11.93 | **0.0026**  0.041  0.15  0.025 |  |
| Rapamycin  500 uM | **[C] 67/4**  [1] 67/4 | **18.18**  18.18 | **+1.56**  -0.44 | **0.61**  0.43 |  |  |  |  |  |  |  |  |
| Rapamycin  1 mM | **[C] 49/13**  [1] 49/13 | **15.89**  15.89 | **-11.23**  -12.98 | **0.0004**  0.0012 |  |  |  |  |  |  |  |  |
| Control  (carbenicillin 4 mM) | **[C] 174/29**  [1] 62/4  [2] 58/13  [3] 52/12 | **26.96**  26.54  26.34  28.13 |  |  |  |  |  |  |  |  |  |  |
| Rapamycin  100 µM  (carbenicillin 4 mM) | **[C] 176/21**  [1] 65/4  [2] 45/10  [3] 66/7 | **17.62**  18.33  16.54  16.79 | **-1.56**  +0.38  -5.16  +5.60 | **0.18**  0.80  0.16  0.24 |  |  |  |  |  |  |  | **<0.0001**  <0.0001  <0.0001  <0.0001 |
| Rapamycin  liposomes  0.5 µM  (carbenicillin 4 mM) | **[C] 196/28**  [1] 61/2  [2] 66/12  [3] 69/4 | **30.04**  30.13  29.36  30.08 | **+11.42**  +13.53  +11.47  +11.57 | **<0.0001**  0.0007  0.0009  0.0001 | **+1.49**  +4.80  +4.74  -6.61 | **0.13**  0.21  0.013  0.24 |  |  |  |  |  | **<0.0001**  <0.0001  <0.0001  <0.0001 |
